# Albuminuria Not Attributable to Diabetic or Hypertensive Kidney Disease: Adult-Onset Minimal Change Disease Presenting With Nephrotic Syndrome in a Woman With Prediabetes and Hypertension

**DOI:** 10.7759/cureus.111816

**Published:** 2026-06-30

**Authors:** Haider Shawket Al-Shakrchy, Zeid Zwain

**Affiliations:** 1 Department of Renal Transplantation, Najaf Nephrology Center, Al-Sader Teaching Hospital, Najaf, IRQ; 2 Department of Diabetes and Endocrinology, Faiha Specialized Diabetes, Endocrine, and Metabolism Center (FDEMC), University of Basrah, Basrah, IRQ

**Keywords:** adult nephrotic syndrome, albuminuria, dyslipidemia, hypertension, minimal change disease, nephrotic syndrome, podocytopathy, prediabetes, proteinuria, steroid-induced diabetes

## Abstract

A 45-year-old woman with long-standing hypertension and premature menopause presented with progressive bilateral pitting leg edema. Initial evaluation showed albuminuria, microscopic hematuria and pyuria, severe mixed dyslipidemia, and prediabetes. Despite optimization of blood pressure and cardiometabolic therapy, edema progressed to the thighs, and urinary protein excretion increased. Renal ultrasonography showed increased parenchymal echogenicity, with scarring of the lower half of the left kidney. Subsequent evaluation revealed hypoalbuminemia and nephrotic-range proteinuria of 8.64 g/day. Autoimmune and viral hepatitis screening was negative, and a right renal biopsy confirmed minimal change disease (MCD)/podocytopathy. Treatment with prednisolone 60 mg/day and diuretic therapy led to rapid clinical remission, with complete resolution of edema and reduction of 24-hour urinary protein to 190 mg/day by May 2025 and 55 mg/day by July 2025. The course was complicated by steroid-induced Cushingoid features and worsening glycemia, requiring temporary addition of glimepiride. By March 2026, the patient remained clinically well with excellent lipid and glycemic control. This case highlights the importance of early nephrology referral and kidney biopsy in adults with progressive albuminuria and edema, even when cardiometabolic comorbidities such as hypertension, prediabetes, and severe dyslipidemia coexist.

## Introduction

Albuminuria refers to increased urinary albumin excretion and is commonly interpreted through the clinical lens of diabetes, hypertension, and cardiometabolic disease. In patients with diabetes or dysglycemia, persistent albuminuria may suggest diabetic kidney disease; however, adult patients may have non-diabetic glomerular disease, particularly when the clinical presentation is atypical. Features such as rapid-onset nephrotic syndrome, active urinary sediment, abrupt change in kidney function, short duration or absence of established diabetes, or disproportionate proteinuria should prompt consideration of non-diabetic kidney disease and timely nephrology evaluation, including kidney biopsy when the result is likely to change management [1-3].

Nephrotic syndrome is a clinical syndrome characterized by heavy proteinuria, hypoalbuminemia, edema, and frequently hyperlipidemia. In adults, the differential diagnosis includes diabetic kidney disease, minimal change disease (MCD), focal segmental glomerulosclerosis, membranous nephropathy, amyloidosis, and other primary or secondary glomerulopathies. Recognizing these alternatives is important because treatment and prognosis differ substantially, and several diagnoses require biopsy-guided immunosuppressive therapy rather than cardiometabolic risk factor management alone [1,4].

MCD is characterized clinically by nephrotic syndrome and pathologically by diffuse podocyte foot-process effacement on electron microscopy, with minimal abnormalities on light microscopy in classic cases. Podocytes are specialized epithelial cells that help maintain the glomerular filtration barrier; injury and effacement of podocyte foot processes increase glomerular permeability to albumin, resulting in marked proteinuria. Although MCD is the most common cause of nephrotic syndrome in children, it accounts for a smaller proportion of adult nephrotic syndrome and therefore requires a high index of suspicion in adults [5,6]. The Kidney Disease: Improving Global Outcomes (KDIGO) guideline recommends glucocorticoids as initial therapy for adult MCD while emphasizing individualized monitoring for treatment toxicity and relapse [1].

This case report describes a middle-aged woman in whom early albuminuria in the setting of prediabetes, hypertension, and severe dyslipidemia initially suggested diabetic or hypertensive kidney disease. However, progression to nephrotic syndrome prompted a kidney biopsy, which revealed steroid-responsive adult-onset MCD. The case highlights the importance of avoiding diagnostic anchoring on cardiometabolic risk factors when albuminuria evolves atypically.

## Case presentation

A 45-year-old woman with a history of hypertension since 2016 presented in February 2025 with bilateral pitting leg edema. Her edema started gradually and increased day by day. At first, it was Grade 3+ and limited to both feet and ankles symmetrically. Her previous antihypertensive therapy had included a transition from amlodipine, candesartan 16 mg, and diltiazem 60 mg, and, in the last year, candesartan/hydrochlorothiazide 16/12.5 mg only. At presentation, HbA1c was 5.9%, consistent with prediabetes, and urine albumin-to-creatinine ratio (ACR) was 177.9 mg/g. The coexistence of prediabetes, hypertension, and albuminuria initially raised concern for early diabetic kidney disease or hypertensive kidney disease. The patient also had severe dyslipidemia with a strong family history of hypercholesterolemia affecting both parents and siblings. Initial lipid profile showed total cholesterol 409 mg/dL, triglycerides 435 mg/dL, high-density lipoprotein cholesterol (HDL-C) 68 mg/dL, very-low-density lipoprotein cholesterol (VLDL-C) 87 mg/dL, and measured low-density lipoprotein cholesterol (LDL-C) 254 mg/dL. Thyroid-stimulating hormone was 2.56 mIU/L (Table 1). General urine examination showed pyuria, bacteriuria, and microscopic hematuria, which were asymptomatic. This is thought to be a simple urinary tract infection, and the patient was prescribed short-term antibiotics (Table 2).

**Table 1:** Baseline measurements Baseline investigations, vital signs, and body measurements.

Parameter	Result	Unit	Reference range
Body mass index (BMI)	27.6	kg/m²	18.5–24.9
Glycated hemoglobin (HbA1c)	5.9	%	<5.7
Random blood glucose	118	mg/dl	70-140
Serum creatinine	0.8	mg/dL	0.4–1.2
Uric acid	3.5	mg/dL	Female: 2–6
Thyroid-stimulating hormone (TSH)	2.56	μIU/mL	0.35–5.1
Ferritin	37.33	ng/mL	Female: 13–232
Total cholesterol	409	mg/dL	<200
Low-density lipoprotein cholesterol (LDL-C)	254	mg/dL	<100
High-density lipoprotein cholesterol (HDL-C)	68	mg/dL	>50
Triglycerides	435	mg/dL	<150
Very-low-density lipoprotein cholesterol (VLDL-C)	87	mg/dL	10–30
Urine albumin	286.8	mg/L	<20
Urine creatinine	161.2	mg/dL	Female: 20–275
Urine albumin-to-creatinine ratio (ACR)	177.9	mg/g	<30
Blood pressure	160/90	mmHg	
24-hour urinary protein	8640	mg/day	< 150

**Table 2:** Baseline urinalysis WBCs: white blood cells count; RBCs: red blood cells count

Parameter	Result
Appearance	Turbid
Albumin	++++
Nitrite	Positive
WBCs	+++
RBCs	+
Bacteria	More than ++++
Specific gravity	1.030
Glucose	Nil
Ketones	Nil

**Figure 1:**
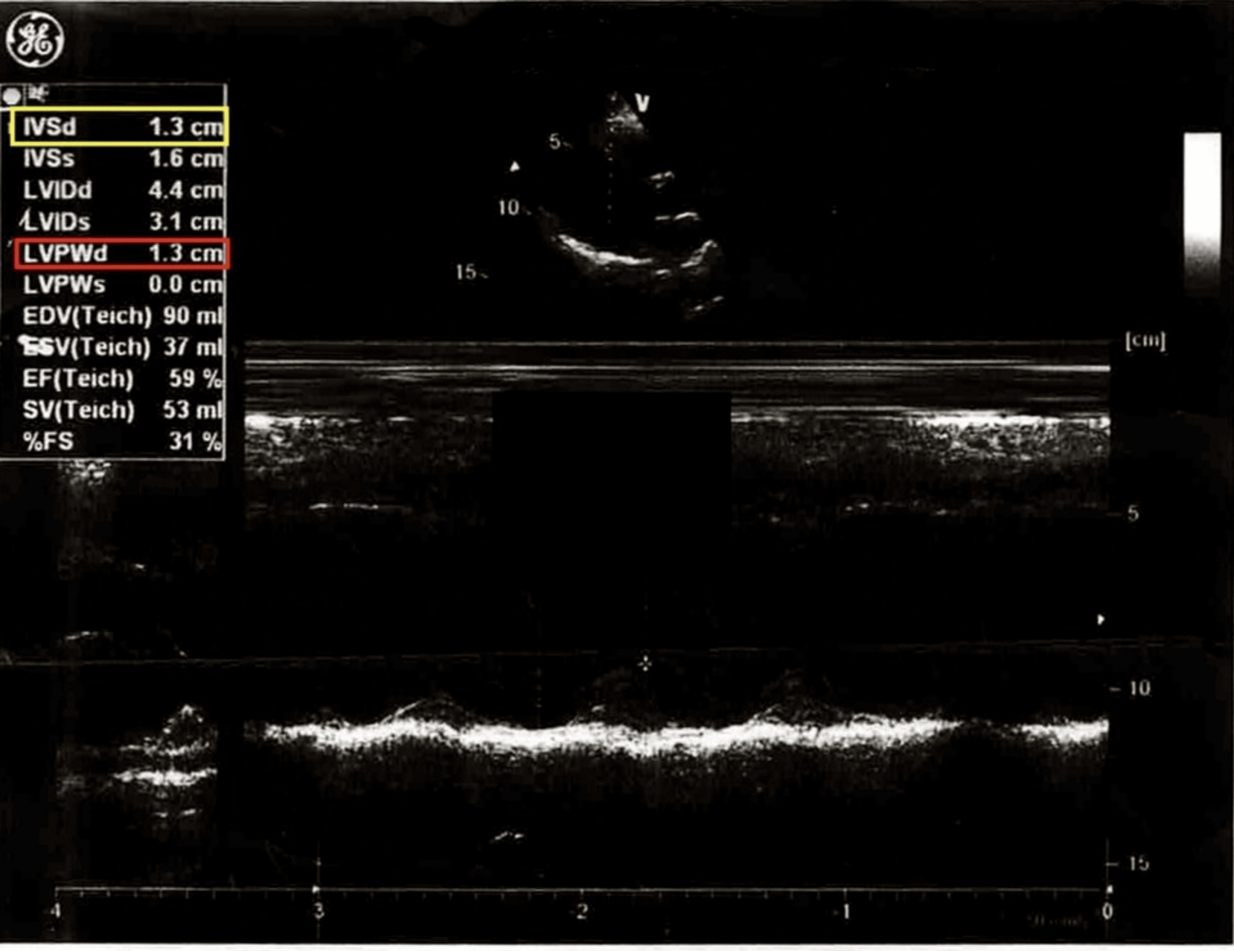
Echocardiography showing left ventricular hypertrophy Yellow rectangle: interventricular septal thickness in diastole (IVSd) of 1.3 cm (normal = 0.6cm - 0.9cm); red rectangle: left ventricular (LV) posterior wall thickness in diastole (LVPWd) of 1.3 cm (normal = 0.6cm - 0.9cm).

Transthoracic echocardiography demonstrated mild concentric left ventricular hypertrophy due to longstanding uncontrolled hypertension, with normal diastolic function, and electrocardiography was normal (Figure 1).

Her reproductive history was notable for early menopause in July 2023 at the age of 43 years, associated with vasomotor symptoms (Table 3).

**Table 3:** Baseline hormonal profile Baseline reproductive hormonal profile indicating early menopause.

Parameter	Result	Unit	Reference range
Follicle-stimulating hormone (FSH)	124.5	mIU/mL	Menopause: 25–134
Luteinising hormone (LH)	120.2	mIU/mL	Postmenopausal: elevated
Estradiol	49.5	pg/mL	Postmenopausal <54
Anti-Müllerian hormone (AMH)	<0.1	ng/mL	2.56–7.13 (optimal fertility)
Testosterone	0.55	ng/mL	Female: 0.1–0.85

Initial treatment included perindopril/indapamide 10/2.5 mg, dapagliflozin 10 mg, rosuvastatin/ezetimibe 40/10 mg, and spironolactone 25 mg. Despite this, edema worsened during March 2025 and extended to both thighs and became Grade 4+. There is no associated dyspnea on exertion or orthopnea. There is no ascites or pleural effusion clinically. Blood pressure was 140/80 mmHg, and ACR increased to 236.7 mg/g (Table 4). Serum creatinine was 0.8 mg/dL, HbA1c 5.7%, random blood glucose 118 mg/dL, ferritin 37.3 ng/mL, alanine aminotransferase 24 U/L, aspartate aminotransferase 29 U/L, alkaline phosphatase 75 U/L, uric acid 3.5 mg/dL, and hepatitis screening was negative. Lipid profile improved but remained abnormal: total cholesterol 216 mg/dL, triglycerides 269 mg/dL, HDL cholesterol 50 mg/dL, VLDL cholesterol 54 mg/dL, and LDL cholesterol 112 mg/dL. Dual-energy X-ray absorptiometry of the spine and hips was normal.

**Table 4:** ACR classification Albumin-creatinine ratio (ACR) classification according to Kidney Disease: Improving Global Outcomes (KDIGO) Guidelines [1].

KDIGO albuminuria category	Description	Urine ACR, mg/g	Urine ACR, mg/mmol
A1	Normal to mildly increased	<30 mg/g	<3 mg/mmol
A2	Moderately increased	30–300 mg/g	3–30 mg/mmol
A3	Severely increased	>300 mg/g	>30 mg/mmol

Diagnostic assessment

**Figure 2:**
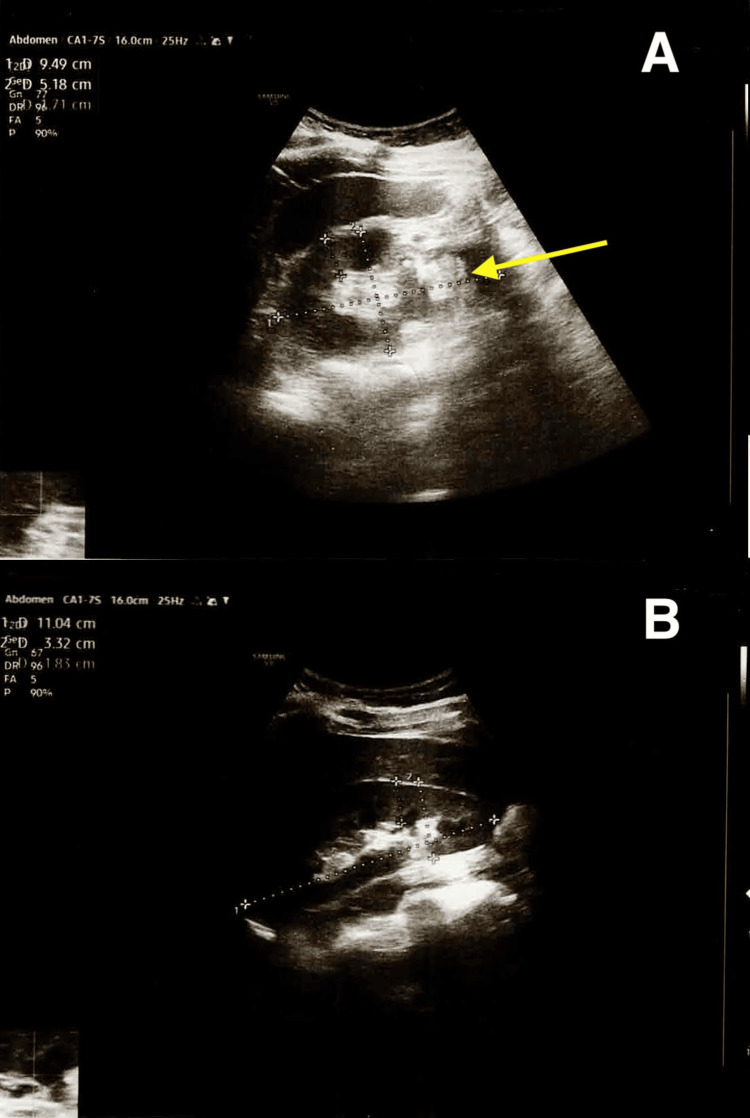
Ultrasonography of the right and left kidneys A: left kidney shows scarring of the lower half (yellow arrow) with increased parenchymal echogenicity; B: right kidney shows mildly increased parenchymal echogenicity with preserved corticomedullary differentiation.

Because the degree and tempo of edema and albuminuria were disproportionate to mild dysglycemia, a nephrology consultation was obtained. Renal ultrasound, kidney biopsy, and 24-hour urine protein quantification were requested. In April 2025, connective tissue disease screening was negative. Kidney ultrasound demonstrated mildly increased parenchymal echogenicity in the right kidney with preserved corticomedullary differentiation, while the left kidney showed scarring of the lower half with increased parenchymal echogenicity (Figure 2).

The clinical picture evolved into nephrotic syndrome. Serum albumin was 2.4 g/dL, creatinine 1.3 mg/dL, urea 51 mg/dL, and calcium 8.4 mg/dL. The 24-hour urine protein level was 8640 mg/day (Table 5). Right kidney biopsy confirmed MCD/podocytopathy. This finding changed the diagnosis from suspected diabetic or hypertensive kidney disease to biopsy-proven adult-onset MCD.

**Table 5:** Reference ranges for 24-hour urinary protein Proteinuria degree according to Kidney Disease: Improving Global Outcomes (KDIGO) Guidelines [1].

Proteinuria category	24-hour urinary protein
Normal	<150 mg/day
Mild proteinuria	150–500 mg/day
Moderate proteinuria	500–3500 mg/day
Nephrotic-range proteinuria	>3500 mg/day (3.5 g/day)

Treatment

After biopsy confirmation of MCD, prednisolone 60 mg/day was started in April 2025. Furosemide 40 mg daily was added for edema management; spironolactone was stopped; and dapagliflozin and sequential estrogen progestin therapy were continued. Close follow-up was maintained jointly between endocrinology and nephrology. Supportive measures included blood pressure optimization, lipid-lowering therapy, diuretic treatment, and monitoring for steroid toxicity. This approach is consistent with adult MCD management, in which corticosteroids remain first-line therapy, while edema, dyslipidemia, blood pressure, and thrombotic risk require parallel supportive care [1,4,6].

Outcome and follow-up

By May 2025 (after about six weeks of starting prednisolone 60 mg/day), bilateral leg edema had completely resolved. The patient developed steroid-induced facial puffiness and dysglycemia, with HbA1c increasing to 7.5%; glimepiride 2 mg was added, and prednisolone tapering was started. Twenty-four-hour urine protein declined dramatically from 8640 mg/day to 190 mg/day, and blood pressure was 120/70 mmHg. At this point, steroid tapering started. The steroid use period was 12 weeks in duration.

In June 2025, renal function improved, with urea 38 mg/dL and creatinine 0.72 mg/dL. Triglycerides decreased to 79 mg/dL, and HDL cholesterol was 65 mg/dL. In July 2025, 24-hour urine protein was 55 mg/day, indicating sustained remission. Perindopril/indapamide was changed to azilsartan 40 mg, while furosemide and dapagliflozin were continued. Blood pressure was 100/70 mmHg.

**Figure 3:**
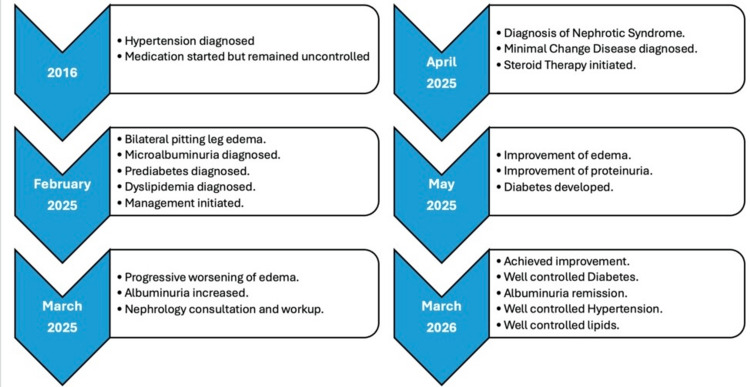
Timeline summary Summarized clinical outcomes and timeline. Figure created using Microsoft PowerPoint 2026 (Microsoft Corporation, Redmond, Washington, United States).

**Figure 4:**
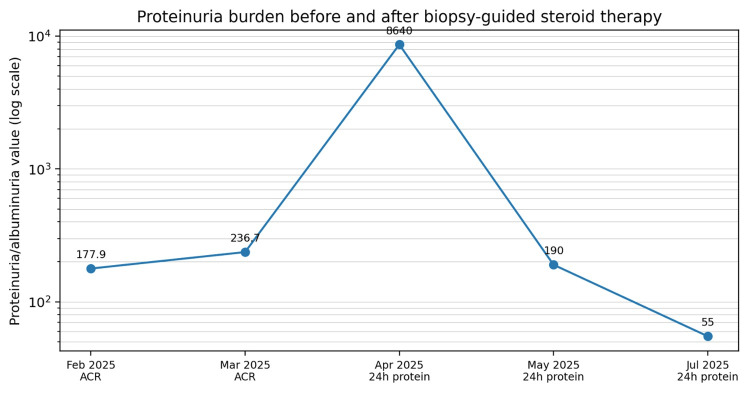
Proteinuria/albuminuria trajectory Albumin-creatinine ratio (ACR) values are shown for February and March 2025, while 24-hour urine protein values are shown from April 2025 onward. The y-axis is logarithmic to display the rapid fall after steroid therapy. Figure created using Python 3 (Python Software Foundation, Fredericksburg, VA) with Matplotlib.

At follow-up in March 2026, the patient was clinically well and comfortable. HbA1c had decreased to 4.5%; total cholesterol was 128 mg/dL, triglycerides 129 mg/dL, HDL cholesterol 49 mg/dL, LDL cholesterol 53 mg/dL, and VLDL cholesterol 26 mg/dL. Her medications were dapagliflozin 10 mg, rosuvastatin/ezetimibe 40/10 mg, azilsartan 40 mg, and sequential estrogen-progestin therapy (Figures 3, 4, and Table 6).

**Table 6:** Chronological detailed timeline ACR: albumin-to-creatinine ratio; BP: blood pressure; DXA: dual-energy X-ray absorptiometry; HDL-C: high-density lipoprotein cholesterol; LDL-C: low-density lipoprotein cholesterol; LVH: left ventricular hypertrophy; MCD: minimal change disease; TG: triglycerides; VLDL-C: very-low-density lipoprotein cholesterol

Time	Event	Investigations	Management
2016	Hypertension diagnosed	-	Sequential antihypertensive exposure: amlodipine, candesartan, diltiazem, candesartan/hydrochlorothiazide
February 2025	Bilateral pitting leg edema; concern for diabetic or hypertensive kidney disease	HbA1c 5.9%; ACR 177.9 mg/g; pyuria and microscopic hematuria; total cholesterol 409 mg/dL; LDL-C 254 mg/dL; TG 435 mg/dL; echo: mild concentric LVH	Perindopril/indapamide, dapagliflozin, rosuvastatin/ezetimibe, spironolactone; menopausal therapy started
March 2025	Edema worsened in both thighs; BP 140/80 mmHg	ACR 236.7 mg/g; creatinine 0.8 mg/dL; HbA1c 5.7%; total cholesterol 216 mg/dL; LDL-C 112 mg/dL; TG 269 mg/dL; DXA normal	Nephrology consultation; renal ultrasound, biopsy, and 24-hour urine protein requested
April 2025	Nephrotic syndrome	24-hour urine protein 8640 mg/day; albumin 2.4 g/dL; creatinine 1.3 mg/dL; connective tissue screen negative; ultrasound: chronic parenchymal changes and left lower-pole scarring; biopsy: MCD/podocytopathy	Prednisolone 60 mg/day started; furosemide added; spironolactone stopped; dapagliflozin and menopausal therapy continued
May 2025	Edema resolved; steroid-induced puffy face and dysglycemia	HbA1c 7.5%; 24-hour urine protein 190 mg/day; BP 120/70 mmHg	Glimepiride 2 mg added; prednisolone tapering started
June 2025	Clinical improvement	Urea 38 mg/dL; creatinine 0.72 mg/dL; TG 79 mg/dL; HDL-C 65 mg/dL	Continued follow-up
July 2025	Remission maintained; BP 100/70 mmHg	24-hour urine protein 55 mg/day	Perindopril/indapamide changed to azilsartan 40 mg; furosemide and dapagliflozin continued
March 2026	Well and comfortable	HbA1c 4.5%; total cholesterol 128 mg/dL; TG 129 mg/dL; HDL-C 49 mg/dL; LDL-C 53 mg/dL; VLDL-C 26 mg/dL	Dapagliflozin 10 mg, rosuvastatin/ezetimibe 40/10 mg, azilsartan 40 mg, and sequential estrogen-progestin therapy

## Discussion

This case illustrates the risk of prematurely attributing albuminuria to diabetic kidney disease when cardiometabolic risk factors are present. The patient had prediabetes rather than established diabetes, and the degree of edema, urinary abnormalities, hypoalbuminemia, and rapid progression to nephrotic-range proteinuria were atypical for early diabetic kidney disease. Studies of kidney biopsy in patients with diabetes or suspected diabetic kidney disease consistently show that non-diabetic kidney disease is common, particularly when the presentation includes nephrotic-range proteinuria, active urinary sediment, short diabetes duration, preserved or atypical renal function, or absence of diabetic microvascular complications [2,3].

MCD is a podocytopathy and a recognized cause of adult nephrotic syndrome. Adults may have a more prolonged time to remission and a higher burden of treatment toxicity than children, making follow-up and steroid-sparing planning important in relapsing or steroid-dependent disease [1,5,6]. The marked fall in proteinuria in this patient after corticosteroid therapy supports the biopsy diagnosis and is consistent with steroid-responsive MCD [1,5,6].

The case also highlights the relationship between nephrotic syndrome and dyslipidemia. Nephrotic syndrome can cause substantial increases in LDL cholesterol, triglyceride-rich lipoproteins, and lipoprotein (a) through increased hepatic lipoprotein synthesis and impaired lipoprotein catabolism [7,8]. Therefore, the severe dyslipidemia seen at presentation may have reflected both familial predisposition and nephrotic physiology. The improvement in lipid parameters after treatment and remission supports the contribution of nephrotic syndrome to the lipid disturbance, although the strong family history remains clinically relevant.

Steroid-induced dysglycemia was another important management issue. The patient entered the clinical course with prediabetes, then developed HbA1c elevation to 7.5% after high-dose prednisolone. This emphasizes the need for proactive glucose monitoring when high-dose glucocorticoids are used, particularly in patients with baseline prediabetes, obesity, or other cardiometabolic risk factors [9]. Her later HbA1c of 4.5% after steroid tapering and clinical recovery suggests that dysglycemia was largely treatment-related and reversible in this case.

Albuminuria should be interpreted as a clinical syndrome rather than a diagnosis. In a patient with prediabetes, hypertension, and dyslipidemia, diabetic kidney disease may appear plausible; however, progressive edema, microscopic hematuria, hypoalbuminemia, and nephrotic-range proteinuria should prompt timely nephrology evaluation and kidney biopsy when the result is likely to change management [1-3].

## Conclusions

The teaching lesson from this case is that progressive albuminuria and edema in a patient with prediabetes, hypertension, and severe dyslipidemia should not always be assumed to represent diabetic or hypertensive kidney disease, especially when the clinical course is atypical. In this patient, worsening bilateral edema, hypoalbuminemia, and nephrotic-range proteinuria were the decisive features that justified nephrology referral and kidney biopsy, despite the presence of cardiometabolic risk factors. The biopsy diagnosis of adult-onset MCD changed management from supportive cardiometabolic therapy alone to glucocorticoid-based treatment, resulting in rapid remission of proteinuria and edema.

Biopsy-confirmed MCD was the main treatable cause of nephrotic syndrome in this patient; however, the case should be interpreted within the broader context of hypertension, prediabetes, dyslipidemia, abnormal renal imaging, and steroid-induced dysglycemia. Recognizing MCD should not erase these background risks, but should prevent diagnostic anchoring to diabetic or hypertensive kidney disease alone.

The case also emphasizes two practical lessons: abnormal urinalysis with pyuria and bacteriuria should be interpreted in a clinical context and should not distract from evaluating significant proteinuria, and high-dose corticosteroid therapy requires proactive monitoring for metabolic toxicity, particularly in patients with baseline prediabetes. This single case does not define the frequency of non-diabetic glomerular disease in similar patients, but it highlights clinical warning signs that should prompt reconsideration of the diagnosis and biopsy-guided management.
